# From crisis response to country control: Restoring agency and sustainability in global health

**DOI:** 10.1371/journal.pmed.1004961

**Published:** 2026-03-03

**Authors:** Ebere Okereke

**Affiliations:** Associate Fellow, Chatham House, The Royal Institute of International Affairs, London, United Kingdom

## Abstract

Global health architecture as we know it is at a structural inflection point. In this Perspective, Ebere Okereke discusses priorities for sustainable progress, including restoring country agency, strengthening national institutions, and securing predictable, sustainable health financing.

## Teaser

Global health is at a structural inflection point. Crisis-driven architectures saved lives but entrenched fragility and dependence. Sustaining progress now requires restoring country agency, strengthening national institutions, and securing predictable, sustainable health financing.

Global health is at a point of structural reckoning. The system that shaped three decades of progress now sits at the edge of its own limits. Financing is tightening, political tolerance for externally driven agendas is waning, and trust between low- and middle-income countries and global institutions is increasingly fragile. At the same time, health threats are becoming more complex, chronic, and deeply intertwined with economic stability, climate risk, and geopolitics. The burden of unmet need remains vast, with recent World Health Organization (WHO) and World Bank monitoring showing stalled progress on universal health coverage and worsening financial hardship driven by out-of-pocket spending [1,2]. This moment is not a temporary disruption; it reflects deeper tensions in how modern global health has been designed and governed.

The current global health architecture was built largely as a response to crises: HIV, polio, malaria, Ebola, COVID-19, etc. Each emergency generated new institutions, financing mechanisms, and delivery platforms designed to move fast and operate at scale in settings with limited state capacity. In that context, speed and control mattered more than integration and ownership [3,4]. The overriding objective (rightly so) was to save lives quickly; however, this often resulted in working around weak systems rather than through and with them.

This model has delivered extraordinary results: Millions of lives have been saved, diseases have been controlled or pushed to the brink of elimination, and coordinated international action has proven its value. Yet the very features that made these initiatives effective in crises have also embedded long-term fragility. Emergency structures, once established, tend to persist. Parallel systems have hardened, vertical programs have expanded [5], and funding flows continue to follow donor priorities rather than domestic plans. Parallel procurement, donor-driven accountability, externally defined targets, and time-bound grants made sense when the alternative was inaction. They make far less sense in a world where countries are expected to manage complex health systems, absorb shocks, and finance their own futures, and where they are increasingly seeking to do so [6].

These dynamics are evident in Ghana, a country often cited as a relative success in health governance. As Ghana has moved through Gavi’s accelerated transition toward self-financing of immunization, long-standing parallel arrangements for vaccine financing, procurement, and reporting have had to be rapidly absorbed into routine government systems. While coverage has largely been maintained, the transition has exposed weaknesses in budget predictability, procurement integration, and institutional coordination [7]. The impact has been increased fiscal stress on the health budget and heightened vulnerability to supply disruptions [7], illustrating how systems designed to operate outside core government structures can leave countries exposed when responsibilities are repatriated.

Similar patterns can also be seen in other areas. In South Africa’s HIV response, external support under the U.S. President’s Emergency Plan for AIDS Relief (PEPFAR) has progressively shifted toward domestic financing and management [8]. Highly effective donor-supported delivery, monitoring, and community outreach platforms proved difficult to fully institutionalize within provincial health systems at scale. The result was not a reversal of political commitment, but uneven integration of functions such as workforce support and data systems, with consequences for service continuity and retention in care in some settings. Again, the fragility lay less in national intent than in the long-term reliance on parallel architectures.

Together, these examples illustrate the core dilemma. This is the central paradox of global health today. Success has created dependence, and speed has come at the cost of agency.

The global health ecosystem was never structured with a clear transition from emergency response to sovereign control. Many flagship global health initiatives were not primarily designed to support countries in taking long-term control of their health systems; they were designed to compensate for its absence. As a result, responsibility and authority have become misaligned. Governments are expected to deliver results without full control over resources or priorities. Accountability flows upward to funders rather than outward to citizens [9]. When financing tightens or global attention shifts, hard-won gains become vulnerable, and national institutions are left exposed.

This tension is not new. Earlier efforts to address aid fragmentation, including sector-wide approaches promoted in the 1990s and 2000s, explicitly sought to align donor financing with national strategies and budgets. While these approaches achieved mixed results [10], they reflected an important recognition: that sustainable progress depends on strengthening country systems rather than perpetually bypassing them.

Health sovereignty has re-entered the global debate in this context. It is sometimes framed as resistance to global cooperation or a retreat into nationalism. That framing is misleading. Sovereignty in health is not about isolation: It is about agency [11]. It refers to a country’s authority over priority-setting, budgetary control, and accountability mechanisms, combined with the institutional capability to deliver on those choices [12]. Without that agency, global health remains inherently fragile.

These concerns are increasingly being acknowledged by governments and global agencies themselves. The Lusaka Agenda, agreed in 2023 by major global health institutions and partner countries, represents an explicit attempt to confront the unintended consequences of fragmentation, parallel systems, and misaligned accountability in the current architecture [13]. By calling for greater alignment behind country-led priorities, stronger national institutions, and reduced transaction costs, the Lusaka Agenda reflects a recognition that sustainability requires a deliberate shift away from crisis-driven exceptionalism toward routine system stewardship.

At a political level, the Accra Reset similarly signals growing impatience among governments with models that deliver short-term results while constraining long-term agency. Its emphasis on mutual accountability, respect for national systems, and a recalibration of power in development partnerships underscores that calls for sovereignty are emerging from states themselves, not only from academic critique [14]. Together, these initiatives suggest that the pressure for reform is now being driven from within the system as well as from those it was designed to support.

Looking ahead, decision-making must move closer to where consequences are felt. This requires a genuine shift from donor-defined priorities toward country-led strategies grounded in domestic political and fiscal realities. It also requires accepting that countries will sometimes make choices external partners would not prefer. That is not a flaw in the system, but the essence of ownership.

Financing models must move away from short-term, project-based funding that undermines long-term system resilience. Predictable financing that supports core functions must become the priority. Co-financing and domestic resource mobilization should be designed in from the start, not introduced only when external actors are ready to exit. Accountability must also be rebalanced. Upward reporting to external funders has dominated global health for too long. Durable accountability runs through parliaments, auditors, civil society, and citizens. That requires sustained investment in governance, transparency, public financial management, and national data systems.

Global institutions will continue to play essential but differentiated roles. Some, such as WHO, are primarily normative and convening. Others, including major financing and delivery platforms, have explicitly operational mandates. The challenge is not to eliminate implementation, but to ensure that external action increasingly reinforces, rather than substitutes for, national systems and accountability.

Ultimately, success must be redefined. Lives saved will always matter. But so should ministries that can plan and execute, budgets that can absorb shocks, and systems that continue to function when external funding declines. If this shift is taken seriously, future success will be evidenced by fewer parallel systems, more predictable domestic financing for core functions, and clearer lines of accountability between governments and their populations. The risk of inaction is equally concrete: repeated cycles of crisis, dependence, and fragility, with diminishing returns on global investment.

Global solidarity remains essential. Shared threats such as pandemics, climate change, and antimicrobial resistance demand collective action. But solidarity cannot substitute for agency, nor can it rely indefinitely on crisis narratives to justify externally concentrated power. The window for deliberate, country-led reform is narrowing.

## Abbreviations

PEPFAR: President’s Emergency Plan for AIDS Relief
WHO: World Health Organization
